# Knowledge and Attitude Toward Sciatica Pain and Treatment Methods Among the Population of Qassim in Saudi Arabia: A Cross-Sectional Study

**DOI:** 10.7759/cureus.64660

**Published:** 2024-07-16

**Authors:** Samar A Alruwaysan, Layan Aljulidan, Mayyaz Alqubays, Maha Alqurzai, Moodhy Aldehsenah, Khadijah I Alburayt, Lama M Aldakhil, Raghad Almarshud, Tameem A Alhomaid

**Affiliations:** 1 College of Medicine, Qassim University, Buraidah, SAU; 2 Family Medicine, Qassim University, Buraidah, SAU; 3 Medicine, Unaizah College of Medicine and Medical Sciences, Qassim University, Unaizah, SAU; 4 Family Medicine, Qassim Health Cluster, Buraidah, SAU

**Keywords:** saudi population, attitude, knowledge, radiating pain, sciatic nerve, sciatica pain

## Abstract

Background*:* Sciatica, a pain radiating along the sciatic nerve, can cause significant suffering and functional limitations. Understanding individual populations' knowledge and attitudes about sciatica pain is crucial for designing targeted interventions and enhancing healthcare delivery, especially in Saudi Arabia. This study aimed to evaluate the knowledge and attitude toward sciatica pain and treatment methods among the population of Al-Qassim in Saudi Arabia.

Methods and materials:This online cross-sectional study was conducted in the Al-Qassim region, Saudi Arabia, using a self-administered questionnaire. The data was analyzed using SPSS software, with numeric data presented as mean ± SD and categorical variables as frequencies and percentages. Correlation analyses included the Chi-squared test and one-way ANOVA.

Results*:* The study received 398 responses, from mostly female (n=305, 76.6%) and Saudi adults aged under 30 (n=248, 62.3%). Most participants sought treatment for sciatica pain from a specialist doctor (n=28, 56.0%) or a general doctor (n=10, 20.0%). Physical therapy was the most common self-treatment method (n=11, 32.4%), followed by painkillers and muscle relaxants (n=10, 29.4%). Knowledge and attitude toward sciatica were generally low (mean score: 3.54 ± 2.61 out of 9), with only 70 (17.6%) showing good knowledge. Most respondents recognized practices like spinal imaging, surgery as a last resort, and exercise/sitting habits as impacting sciatica outcomes. Traditional therapies like massage, cupping, acupuncture, and cautery were considered beneficial. Educational level significantly impacted knowledge scores, with higher mean scores among postgraduate education holders and bachelor's degree holders (mean scores: 4.06 ± 2.48 and 3.98 ± 2.53, respectively). Age, gender, occupation, nationality, and region showed no significant differences in mean knowledge scores. Attitude scores were similar across sociodemographic spectra, with younger respondents having slightly more positive attitudes.

Conclusion*:* The study showed poor knowledge, influenced by education levels, and neutral attitudes about sciatica among residents of Al-Qassim. Therefore, educational programs and engagement of healthcare stakeholders are recommended to raise awareness and improve knowledge and attitudes.

## Introduction

Sciatica, defined as radiating pain along the sciatic nerve, can cause significant suffering and functional limitations, affecting mobility, productivity, and general well-being [1]. The lifetime prevalence of this condition is believed to be between 13% and 40%, with the vast majority of cases resolving spontaneously with analgesics and physiotherapy [2,3]. The most prevalent cause of radicular leg pain is disc herniation caused by age-related degenerative changes and, in rare cases, trauma [4], but lumbar stenosis and, less frequently, tumors are other possible causes. Physical exercise raises the likelihood of recurring sciatic symptoms for patients with past sciatic symptoms, while the likelihood decreases in those with no prior symptoms [1].

Recent evidence showed that while the adult population's attitude about sciatica pain was largely positive in Saudi Arabia, their knowledge was inadequate, with 60.1% scoring 3.8 out of 10. Individuals with a bachelor's or higher degree, as well as those who live in cities, were found to have better knowledge and attitudes concerning sciatica pain than those living in rural regions [5]. Moreover, it was found that orthopedic surgeons or neurosurgeons were the most common sources of information about sciatica, while pain relievers were commonly utilized for self-treatment (30.8%) [5]. Cultural norms were also suggested to influence perceptions of pain and treatment-seeking behaviors [6,7].

While previous studies explored sciatica in various global contexts and few studies specifically address this issue in Saudi Arabia [8-11], there is still a lack of studies exploring knowledge and attitudes toward sciatica pain in the Qassim region, despite the presence of pain management facilities, such as KKT Orthopedic Spine Center in Qassim, which offers pain therapy for people with spinal problems, such as sciatica [12]. Therefore, this study aimed to evaluate the knowledge and attitude toward sciatica pain and treatment methods among the population of Al-Qassim in Saudi Arabia. Our study’s findings will bridge the gap between scientific understanding and community perspectives and inform targeted interventions, especially in the Qassim region and Saudi Arabia as a whole, contributing to the improvement of healthcare outcomes and quality of life for people living with sciatica in Saudi Arabia.

## Materials and methods

This was an online cross-sectional study carried out in the Al-Qassim region in Saudi Arabia between August and October 2023. Our study included all adults (aged 18 and above) who had radiating leg pain, consented to participate in the study, and were residents of the Al-Qassim region.

The sample size was estimated using the following formula, considering a margin of error of 5% and a confidence interval of 95%. n= Z^2^(1-P)P/e^2^, where n=sample size, Z= 1.96: z-score at 95% confidence interval, p=50%: sample proportion, assuming an average response for most of the questions of 50%, and e=5%: The margin of error at a 95% confidence interval. The minimum sample size (n) was 385, and to compensate for any possible data loss and unresponsiveness, the total sample size used was 400 participants. We used a convenience sampling technique to select participants.

For data collection, we used a valid pre-tested structured online questionnaire from a previously published article. The online Arabic questionnaire was added to Google Forms and distributed electronically via social media apps (WhatsApp and Twitter) together with the invitation letter and informed consent form. The invitation letter contained a description of the study, its objectives, participants’ rights, and contacts for further inquiries. The submission of the completed questionnaire was done after submitting the signed consent form.

The questionnaire inquired about demographic information and general information about knowledge, awareness, and attitude toward sciatica pain. All questions were mandatory, and they were either multiple-choice questions with single or multiple answers that applied. 

The questionnaire was pre-tested in a pilot study on 25 participants, and results were included in the main study. The pilot test results were used for modifications to ensure clarity and easy understanding of the questions. Three experts in neurology and rheumatology revised and validated the questionnaire and a Cronbach alpha reliability test showed that the coefficient was 0.83, indicating a good reliability. 

The data were transferred to IBM SPSS Statistics for Windows, Version 26 (Released 2019; IBM Corp., Armonk, New York, United States) for statistical analyses. Numeric data were presented as mean ± standard deviation (SD) or median and range according to each variable's type of distribution. For categorical variables, frequencies and percentages were used for data presentation. Depending on the types of variables, the chi-squared test and one-way ANOVA were used for correlation analyses.

The Buraydah Central Hospital Institutional Review Board approved this study. The questionnaire was anonymous to ensure confidentiality, and the study was conducted according to the WMA Declaration of Helsinki.

## Results

Participants’ characteristics

This study received 398 responses, predominantly female (n=305, 76.6%) and young Saudi adults under 30 years old (n=248, 62.3%). A quarter of the participants were unemployed (n=101, 25.4%). Less than half were students (n=190, 47.7%). Almost all participants resided in central Al-Qassim (n=390, 98%). Table 1 shows the demographic characteristics of the participants in the study. 

**Table 1:** Demographic characteristics of the study sample

Socio-demographic characteristics	N	%
Nationality		
Saudi	395	99.2%
Non-Saudi	3	0.8%
Age category (years)		
Less than 18	17	4.3%
18-30	248	62.3%
31-50	100	25.1%
Above 50	33	8.3%
Gender		
Female	305	76.6%
Male	93	23.4%
Educational level		
Primary school	14	3.5%
Secondary school	178	44.7%
Bachelor's degree	188	47.2%
Higher education	18	4.5%
Occupation		
Student	190	47.7%
Office work	39	9.8%
Fieldwork	17	4.3%
Unemployed	101	25.4%
Education sector	31	7.8%
Healthcare practitioner	20	5.0%
Place of residence		
Central region	390	98.0%
Other region	8	2.0%

**Figure 1:**
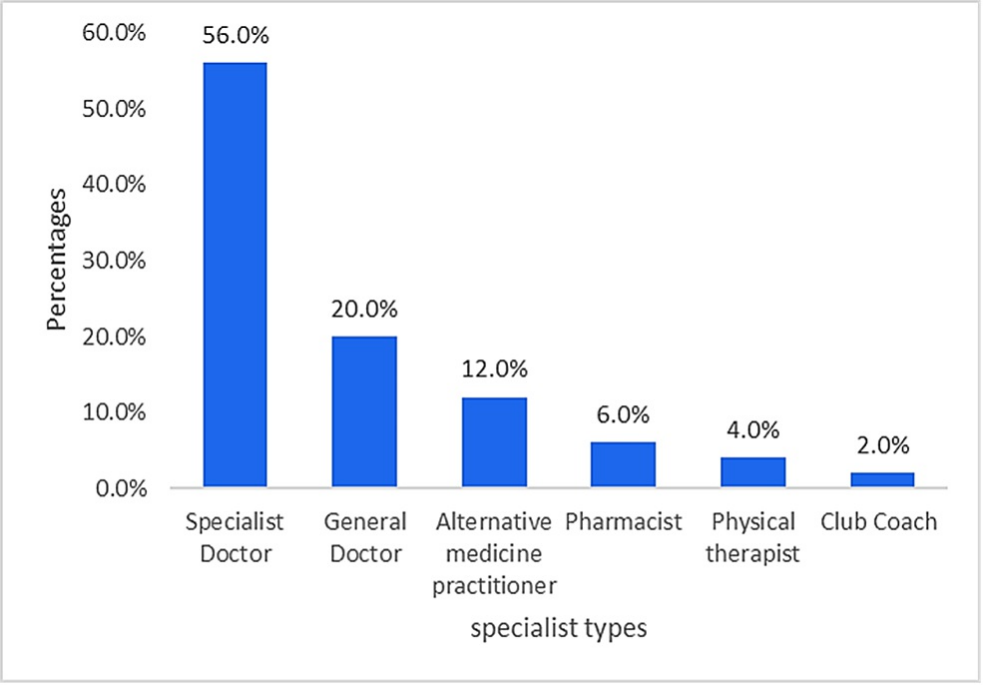
The specialist who provided advice after experiencing sciatica pain

**Figure 2:**
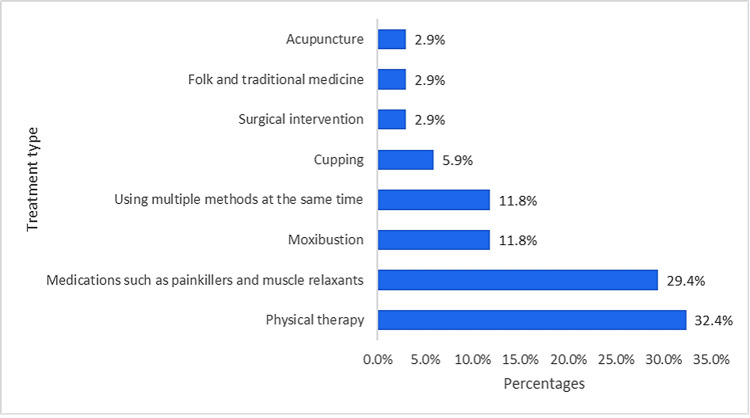
Self-treatment of sciatica

Figure 1 illustrates that among participants who experienced sciatica pain, most sought treatment from a specialist doctor (n=28, 56.0%) or a general doctor (n=10, 20.0%). Only a tiny proportion followed an alternative medicine practitioner (traditional healer) (n=6, 12.0%), pharmacist (n=3, 6.0%), physiotherapist (n=2, 4.0%), or gym coach (n=1, 2.0%). Moreover, the most common self-treatment method for sciatica pain reported by participants was physical therapy (n=11, 32.4%), followed by painkillers and muscle relaxants (n=10, 29.4%). Furthermore, 11.8% (n=4) equally used moxibustion and multiple methods simultaneously. Few other methods, such as cupping, acupuncture, or traditional medicine, have been used (Figure 2).

Epidemiology of sciatica

Table 2 shows the demographic differences between participants diagnosed with sciatica and those who were not. A statistically significant differences in sciatica diagnoses were found among age categories (p< 0.001). Participants aged 31-50 years were more likely to be diagnosed with sciatica compared to other age groups. 

**Table 2:** Sociodemographic characteristics of participants in accordance with sciatica pain The p-value is calculated by a chi-square test. * Significant at p< 0.01

Socio-demographic characteristics	Diagnosed with sciatica pain		p-value
	No, N (%), n= 368	Yes N (%), n= 30	
Nationality			
Saudi	366 (99.5%)	29 (96.7%)	0.21
Non-Saudi	2 (0.5%)	1 (3.3%)	
Age category (years)			
Less than 18	16 (4.3%)	1 (3.3%)	0.001**
18-30	239 (64.9%)	9 (30.0%)	
31-50	85 (23.1%)	15 (50.0%)	
Above 50	28 (7.6%)	5 (16.7%)	
Gender			
Female	284 (77.2%)	21 (70.0%)	0.373
Male	84 (22.8%)	9 (30.0%)	
Educational level			
Primary school	12 (3.3%)	2 (6.7%)	0.343
Secondary school	166 (45.1%)	12 (40.0%)	
Bachelor's degree	175 (47.6%)	13 (43.3%)	
Higher education	15 (4.1%)	3 (10.0%)	
Occupation			
Student	182 (49.5%)	8 (26.7%)	0.137
Office work	34 (9.2%)	5 (16.7%)	
Fieldwork	16 (4.3%)	1 (3.3%)	
Unemployed	92 (25.0%)	9 (30.0%)	
Education sector	26 (7.1%)	5 (16.7%)	
Healthcare practitioner	18 (4.9%)	2 (6.7%)	
Place of residence			
Central region	360 (97.8%)	30 (100.0%)	0.999
Other regions	8 (2.2%)	0 (0.0%)	

Knowledge and attitude toward sciatica pain

Table 3 shows the knowledge and attitude toward sciatica pain among the participants in the study. First, regarding knowledge, the mean score was low (3.54 ± 2.61 out of 9), indicating generally poor knowledge of sciatica. Only 70 (17.6%) demonstrated good knowledge, and the most well-known facts were the causes like herniated discs (n=265, 66.6% correct) and risk factors like age, weight, and prolonged sitting (n=237, 59.5% correct). However, gaps existed in identifying sciatica symptoms (n=157, 39.4% correct), treatments like physiotherapy and medications (n=119-143, 29.9-35.9% correct), and prognosis/disability risks (n=58-88, 14.6-22.1% correct).

**Table 3:** Knowledge and attitude toward sciatica pain (n= 398) NSAIDs: Non-steroidal anti-inflammatory drugs; SD: Standard deviation; CT/MRI: Computed tomography/magnetic resonance imaging

Knowledge statement	Correct answer N (%)
(1) The most distinctive sign of sciatica is pain that radiates from your lower back into the back or side of your legs	157 (39.4%)
(2) Pain, numbness, tingling sensation extending from the lower back down to the toes, and weakness of leg/foot muscles are symptoms of sciatica	247 (62.1%)
(3) The most common cause of sciatica is a herniated vertebral disc, which often occurs with age	265 (66.6%)
(4) Age, weight, nature of work, and prolonged sitting are risk factors for sciatica	237 (59.5%)
(5) Sciatica is thought to be preventable and it may not recur	95 (23.9%)
(6) Physiotherapy and steroid injections are methods to reduce/treat sciatica	143 (35.9%)
(7) NSAIDs and muscle relaxants are methods to reduce/treat sciatica	119 (29.9%)
(8) People with sciatica should avoid movement as it may cause more injury	88 (22.1%)
(9) Having sciatica may mean you will end up with movement disability	58 (14.6%)
Knowledge score (mean ± SD)	3.54 ± 2.61
Level of knowledge	
Poor	247 (62.1%)
Moderate	81 (20.4%)
Good	70 (17.6%)
Attitude statement	Mean ± SD
(1) The severity of pain varies from mild to very severe and it intensifies when sneezing or coughing or after prolonged sitting	3.59 ± 0.876
(2) Spinal CT/MRI can diagnose sciatica	3.75 ± 0.953
(3) Surgical intervention is the last method to relieve sciatica	3.43 ± 0.963
(4) Drinking turmeric and cinnamon mixed with warm milk can reduce/treat sciatica pain	2.85 ± 0.910
(5) Mustard oil massage can reduce/treat sciatica pain	3.10 ± 0.946
(6) FASD (blood-letting) is one of the most effective ways in reducing/treating sciatica	3.03 ± 0.953
(7) Moxibustion and cautery can reduce/treat sciatica pain	2.99 ± 1.043
(8) Acupuncture can reduce/treat sciatica pain	3.07 ± 0.899
(9) Cupping therapy can reduce/treat sciatica pain	3.27 ± 0.882
(10) Regular exercising and proper sitting can significantly contribute to back protection	4.11 ± 0.869
(11) Traditional therapy is more effective than medical intervention in treating sciatica	2.91 ± 1.058
Attitude score (mean ± SD)	36.12 ± 5.52
Level of attitude	
Negative	19 (4.8%)
Neutral	324 (81.4%)
Positive	55 (13.8%)

On the other hand, the overall level of attitudes was neutral, with a mean score of 36.12 out of 55. Most respondents correctly recognized that practices like spinal imaging (mean: 3.75 ± 0.95), surgery as a last resort (mean: 3.43 ± 0.96), and exercise/sitting habits (mean: 4.11 ± 0.87) could impact sciatica outcomes. However, traditional therapies, such as massage (mean: 3.10 ± 0.95), cupping (mean: 3.27 ± 0.89), acupuncture (mean: 3.07 ± 0.9), and cautery (mean 2.99), were considered beneficial by many.

**Figure 3:**
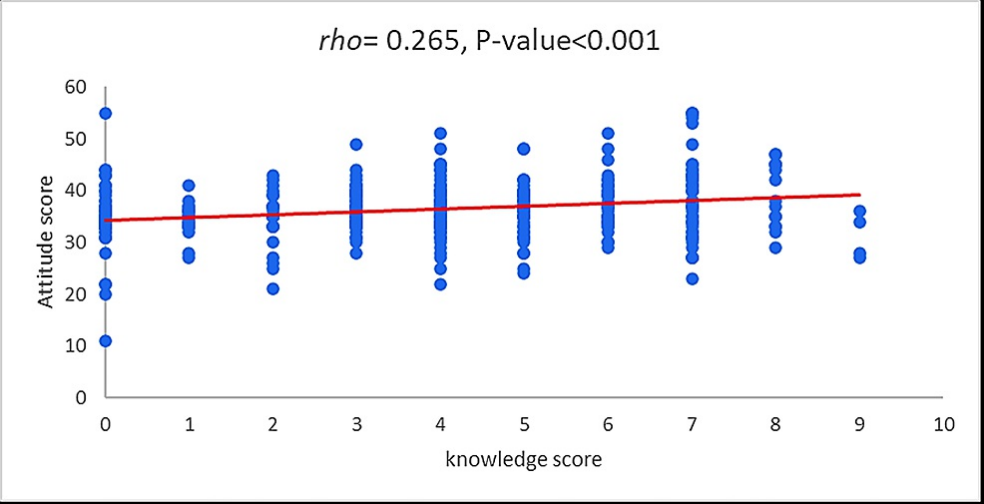
Correlation (Spearman rho) between the knowledge score and attitude score

Figure 3 illustrates the relationship between the knowledge and attitude score. A significant positive correlation was observed between the knowledge and attitude scores, with a Sperman-Rho correlation of 0.265 (p< 0.001).

Patient's characteristics and their knowledge and attitude toward sciatica pain

Table 4 shows how knowledge and attitudes regarding sciatica vary across different sociodemographic groups within the study sample. For knowledge scores, the educational level had a significant impact (p= 0.002), with higher mean scores among bachelor's degree holders (mean: 3.98 ± 2.53) and those with postgraduate education levels (mean: 4.06 ± 2.48) compared to secondary school (mean: 3.14 ± 2.65) and primary school levels among participants (mean: 2.07 ± 2.43). This indicates that higher education is associated with improved sciatica knowledge. Meanwhile, age, gender, occupation, nationality, and region showed no statistically significant differences in mean knowledge scores (p> 0.05). However, some variations were observed. For example, older adults above 50 years scored higher (mean: 4.09 ± 2.78) than younger groups , and employed persons in fieldwork had the highest knowledge scores (mean: 4.94 ± 2.14). 

**Table 4:** Differences in knowledge and attitude scores based on participant sociodemographic characteristics (n= 398) * Significant at p< 0.01; SD: Standard deviation

Socio-demographic characteristics	Knowledge			Attitude		
	Score (9) Mean ± SD	Z/H-test	p-value	Score (55) Mean ± SD	Z/H-test	p-value
Nationality						
Saudi	3.54 ± 2.61	Z = -0.176	0.860	36.1 ± 5.54	Z = -0.663	0.508
Non-Saudi	3.33 ± 3.51			37.7 ± 4.2		
Age category (years)						
Less than 18	3.24 ± 2.97	H = 1.45	0.693	37.4 ± 5.10	H = 3.85	0.278
18-30	3.58 ± 2.60			35.7 ± 5.64		
31-50	2.60 ± 3.63			36.8 ± 5.38		
Above 50	4.09 ± 2.78			36.3 ± 5.25		
Gender						
Female	3.47 ± 2.63	Z = -0.989	0.323	36.2 ± 5.41	Z = -0.750	0.454
Male	3.77 ± 2.58			35.9 ± 5.92		
Educational level						
Primary school	2.07 ± 2.43	H = 14.6	0.002**	38.7 ± 5.86	H = 4.58	0.205
Secondary school	3.14 ± 2.65			36.3 ± 5.64		
Bachelor's degree	3.98 ± 2.53			35.8 ± 5.32		
Higher education	4.06 ± 2.48			35.6 ± 6.05		
Occupation						
Student	3.59 ± 2.55	H = 6.79	0.237	35.7 ± 5.36	H = 5.30	0.385
Office work	3.36 ± 2.88			36.2 ± 5.97		
Fieldwork	4.94 ± 2.14			37.4 ± 3.89		
Unemployed	3.32 ± 2.75			36.5 ± 5.41		
Education sector	3.32 ± 2.41			36.0 ± 6.32		
Healthcare practitioner	3.65 ± 2.52			36.7 ± 6.77		
Place of residence						
Central region	3.56 ± 2.62	Z = -1.33	0.184	36.1 ± 5.56	Z = -0.396	0.692
Other regions	2.38 ± 2.07			35.4 ± 3.25		

No significant demographic variations were observed for attitude scores (p> 0.05), though minor differences existed. For instance, younger respondents aged below 18 had slightly more positive attitudes (mean: 37.4 ± 5.10) compared to other groups. These findings indicate that the education level predicts better sciatica knowledge, while attitudes appear similar across sociodemographic spectra.

## Discussion

The findings of this study provide valuable information on the population's knowledge, attitudes, and treatment-seeking behaviors about sciatica pain in Qassim, Saudi Arabia.

The study found that a large proportion of participants sought therapy from specialized doctors or general practitioners, showing a dependence on medical professionals to manage sciatica. This is consistent with prior research, which found that people frequently seek assistance from healthcare providers because sciatica is so severe and can lead to significant disabilities [2]. Another study found that patients with sciatica are commonly managed at the primary care level but a small proportion is referred to secondary or tertiary care levels for further management, which might include surgery [13]. Furthermore, the preference for physical therapy over other self-treatment approaches among participants emphasizes the importance of rehabilitation in sciatica management, which is consistent with research demonstrating the benefit of physical exercises in pain reduction and function improvement [14-16]. A randomized controlled trial showed that physiotherapy modalities using exercises significantly (p<0.001) reduced pain between the physical therapy group and the control group after three months and one year of treatment [14]. However, it is worth noting that a small proportion of participants in our study sought alternative medicine practitioners or self-medicated with opioids and muscle relaxants. This shows a heterogeneous approach to sciatica management, which may be influenced by cultural beliefs, accessibility, and perceived efficacy of standard treatments. Evidence shows that treatment methods and practices vary amongst individuals based on their cultural background, social situation, and religious views [8]. Some alternative therapies, such as moxibustion, acupuncture, etc., have demonstrated promising benefits in treating sciatica symptoms [17,18], which aligns with our findings that they were also considered effective by some of our participants. However, there is still a need for more studies to further explore the safety and benefits of alternative therapy modalities for sciatica. The use of such methods by fewer participants in this study suggests a preference for conventional medical interventions. The population should be encouraged to seek help from professionals who can rule out life-threatening causes, such as disc herniation and tumors, and orient appropriate interventions other than jumping to alternative therapies, which are not widely accepted or proven in the scientific community and by some guidelines. For instance, acupuncture is not recommended for sciatica, and NICE guidelines advise against acupuncture, traction, and electrotherapies for back pain with or without sciatica [13]. Moreover, acupuncture, extraforaminal glucocorticoid injection, paracetamol, non-steroidal anti-inflammatory drugs, and opioids are all discouraged by Danish guidelines [19]. Clinical recommendations from the United Kingdom, the United States, and Denmark encourage exercise but do not specify whether one sort of exercise is preferable to another [13,19].

The study identified a knowledge gap in sciatica among Qassim's residents. With a low mean knowledge score and a small proportion of participants displaying good knowledge, there is an obvious need for educational programs to raise awareness about sciatica, its symptoms, risk factors, and treatment modalities. This finding is consistent with previous studies suggesting a lack of public understanding of sciatica and its treatment [8,20]. Our findings also align with a previous study conducted in Saudi Arabia nationwide that showed that the mean knowledge score was 3.8, and most had poor knowledge (60.1%) [8], which is similar to our study’s findings. Another study showed that the mean knowledge of sciatica causes and the nature of pain and treatment was 3.6 [5]. This underlines the necessity of targeted interventions to address this inadequate knowledge in Saudi Arabia. However, another study conducted in Saudi Arabia found the knowledge of sciatica to be moderate [9]. By addressing misunderstandings and giving factual information through public health campaigns and patient education materials, the Saudi population can be empowered to make educated health decisions and seek appropriate care as soon as possible. It was shown that patients reported a desire for credible information, citing it as a key step in comprehending their illness prior to therapy, and healthcare providers, especially orthopedics and neurosurgeons, were key to informing the population [8,20].

Despite their inadequate knowledge, participants maintained a neutral attitude regarding sciatica, acknowledging the relevance of evidence-based therapies such as spine imaging and exercises for sciatica management. Our findings about neutral attitudes align with what was previously reported across Saudi Arabia [8]. Our findings are also similar to those of another previous study, showing that the attitudes were sufficient despite the inadequate knowledge of sciatica among the Saudi population [5]. However, the persistence of misunderstandings about unproven traditional and alternative therapies emphasizes the significance of encouraging evidence-based practices while discouraging ineffective or potentially hazardous treatments. Healthcare practitioners can play an important role in teaching patients about the risks and advantages of various treatment options, facilitating collaborative decision-making, and encouraging adherence to prescribed management measures [21,22]. Previous research confirmed the role of healthcare providers in raising awareness by showing that the most common information sources about sciatica are orthopedics or neurosurgeons [8].

The study found significant demographic differences in knowledge levels, with higher education levels being related to a better knowledge of sciatica. This finding is consistent with prior research that has shown the impact of education on health literacy and healthcare-seeking behaviors [23,24]. Similar to our findings, previous studies also reported that having a bachelor’s and higher degrees was positively associated with high knowledge of sciatica [5,8,9], which confirms the evidence that high education leads to better health literacy. Therefore, efforts to raise sciatica knowledge should focus on persons with lower educational levels, using personalized educational programs and easily accessible resources.

Interestingly, while attitudes toward sciatica did not change significantly among demographic groups, there were slight disparities. Younger respondents had slightly more positive attitudes, indicating a possible age difference in healthcare ideas and preferences, as shown by previous research [25]. In Saudi Arabia, previous studies showed that adults with comorbidities residing in cities had better attitudes and knowledge of sciatica [5,8], which might have been due to their frequent interaction with healthcare providers for their diseases. However, more research is needed to investigate the underlying causes of these variations identified by our study, though not statistically significant, and guide targeted interventions to increase positive attitudes toward sciatica management across all age groups.

This study has some limitations to consider. This study was an online cross-sectional study, with the inability to establish causality or change in variables over time, and cannot establish cause-and-effect relationships between variables. Cross-sectional studies may be susceptible to bias, selection bias, measurement bias, or confounding variables, impacting the validity of results. Its online nature is also prone to selection bias as participants with no access to the internet are left out. The self-administered questionnaire nature also exposes the study to underreporting and overreporting risks. The questionnaire did not inquire about some therapies, such as home therapy, exercise, YouTube tutorials, and spa treatments, limiting adequate representation. Moreover, the convenience sampling technique used is also prone to biases and limited representativeness. Therefore, extensive conducted nationwide with a complex research methodology mixing quantitative and qualitative designs is recommended to further explore sciatica, associated factors, and treatments and deeply provide answers to how and why in order to inform health innervations to address the issue. To achieve these, future studies encompassing all therapy domains should use longitudinal or time-lagged designs to track changes in variables over time, establishing temporal precedence between exposures and outcomes, employ case-control or cohort studies for causal inference, consider factors like reverse causality, which may influence association direction, and address selection bias due to sampling methods and lack of follow-up.

## Conclusions

This study generally showed poor knowledge and neutral attitudes among residents of Al-Qassim, with the knowledge being influenced by education levels. These findings highlight the need for educational programs to promote knowledge about sciatica and evidence-based treatments among the Qassim population. Healthcare providers should take a proactive approach to refuting misunderstandings, advocating viable treatment options, and encouraging patients to consult professionals. Healthcare stakeholders can also be engaged to improve care quality and reduce the impact of this debilitating condition on people’s lives as a whole by addressing information gaps and developing positive attitudes about sciatica management.
